# Supplementary material for: Epidemic risk of arboviral diseases: Determining the habitats, spatial-temporal distribution, and abundance of immature Aedes aegypti in the Urban and Rural areas of Zanzibar, Tanzania
Source: PLoS Negl Trop Dis. 2020 Dec 7;14(12):e0008949. doi: 10.1371/journal.pntd.0008949 (PMC7746278; doi:10.1371/journal.pntd.0008949)
Supplement: S1 Table — aPercent of all mosquitoes by setting or season. (PDF) [file pntd.0008949.s001.pdf]

S1 Table. Distribution of mosquito genera/species by season in rural and urban areas of Zanzibar.

	Ae. aegypti	*Ae. simpsoni*	*Culex*	*An. gambiae s.l.*	*Eretmapodites*	
	N (%[a])	N (%[a])	N (%[a])	N (%[a])	N (%[a])	Total
**Rural**	8,642 (82.6)	6 (0.1)	1,590 (15.2)	9 (0.1)	207 (2.0)	10,454
Wet	5,795 (85.1)	0 (0.0)	798 (11.8)	7 (0.1)	207 (3.0)	6,807
Dry	2,847 (78.1)	6 (0.2)	792 (21.7)	2 (0.1)	0 (0.0)	3,647
**Urban**	5,029 (64.3)	0 (0.0)	2,794 (35.7)	0 (0.0)	0 (0.0)	7,823
Wet	3,379 (92.9)	0 (0.0)	259 (7.1)	0 (0.0)	0 (0.0)	3,638
Dry	1,650 (39.4)	0 (0.0)	2,535 (60.6)	0 (0.0)	0 (0.0)	4,185
Total	13,671 (74.8)	6 (0.03)	4,384 (24.0)	9 (0.05)	207 (1.2)	18,277

[a] Percent of all mosquitoes by setting or season.
