# Supplementary material for: Epidemic risk of arboviral diseases: Determining the habitats, spatial-temporal distribution, and abundance of immature Aedes aegypti in the Urban and Rural areas of Zanzibar, Tanzania
Source: PLoS Negl Trop Dis. 2020 Dec 7;14(12):e0008949. doi: 10.1371/journal.pntd.0008949 (PMC7746278; doi:10.1371/journal.pntd.0008949)
Supplement: Attachment — Submitted filename: Response to Reviewers file.docx [file pntd.0008949.s002.docx]

**Cover letter regarding revision of the manuscript entitled: “Epidemic risk of arboviral diseases: determining the habitats, spatial-temporal distribution and abundance of immature *Aedes aegypti* in the Urban and Rural areas of Zanzibar, Tanzania” submitted to PLoS NTD journal.**

We highly appreciate the constructive comments, suggestions, and corrections made by both reviewers, and journal Editor, and would like to acknowledge their time and efforts spent on our manuscript. In this letter, we have point-by-point addressed the comments from the reviewers and Editor, as received from the journal Editor. Our response is written in blue color font.

**Editor’s comments**

1. Lines 25, 58, 59, 60, 63 and others: Although commonly used, mosquitoes do NOT breed in water. Breeding has a reproductive/sexual connotation. Mosquito larvae (and pupae) develop in water. I would use “larval habitats” in place of “breeding habitats” throughout the manuscript.

We have removed “breeding habitats” and replaced with “larval habitats” throughout the manuscript text

2. Line 95: main hospital in 2013 (Ali Mohammed, unpublished data). Who is Ali Mohammed? Do you have permission to site his work, where does he work, why is he not cited in the acknowledgements?

Mr Ali Mohammed is a colleague and a medical laboratory personnel at the State University of Zanzibar. He conducted a study on etiologic agents of fever of unknown origin as part of his graduate studies at Muhimbili University of Health and Allied Sciences, Dar es Salaam, Tanzania. Although Mr Mohammed had granted his permission to cite his findings, these have now been published in the Journal of Community Health, to which we refer in the revised manuscript (Ref 20), - Lines 102 - 103.

3. Lines 108-110: Bed nets tend to protect people at night, but Ae. aegypti is a day-biting mosquito.

The statement was meant to describe the ongoing vector control interventions in Zanzibar, which focus exclusively on a nocturnal biting malaria vector. However, for clarity we have inserted …... ‘with the aim of eliminating malaria. Both methods are hampered by the development of insecticide resistance [26], while LLINs are ineffective against diurnal *Aedes* mosquitoes. Nonetheless,….. – Lines 117 – 119.

4. Lines 140-144: Is the rainfall amount really accurate to 0.1 mm? I would tend to round all of these off to the nearest whole mm. When someone claims a degree of accuracy not supported by the data, readers tend to question all of the data.

The rainfall amounts have been rounded to the nearest whole numbers – Lines 150 – 152

Please note that the reported meteorological data is only available on request from the local meteorological office and was provided to me by the Director of Tanzania meteorological Authority following personal communication and a formal letter of request. Therefore, the reference ‘unpublished data’ was replaced with ‘personal communication’ Lines 153 – 154 and a letter of support is submitted through the Editorial manager.

5. Lines 144-145 (and Figure 1): This may be a little confusing. Here it says that, “…the urban and West districts that make up the Urban-West region of Unguja.” For those people not familiar with the Mjini and Magharibi districts within the Mjini Magharibi Region, they may not realize that the Urban-West Region is made up of two districts, the Urban District and the West District. Unfortunately, looking at Fig. 1 does not help as one cannot see which parts of the Region are in which district. It might be clearer if the two districts were shown in different “patterns” and the study areas highlighted.

We have clarified the statement (Lines 155 – 156) and used distinct colours to mark the two districts of the Region in Fig 1.

6. Lines 146-149: Minor, but these data are from the 2012 census, i.e., several years before the actual study. Again, is it really important that 593,678 people lived there 6 years before the study, or would it be better to say that according to a 2012 census [29], the population was about “594,000” or “nearly 600,000?

The suggested changes inserted – Lines 157 – 160.

7. Line 175: Minor, but “water holding containers…” should be “water-holding containers…”

“Water-holding containers” inserted – Line 188 and throughout the manuscript

8. Line 197: Minor, but “two one-week…” should be “two 1-week……” Similarly, it should be “3 to 5 days” or 3-5 days” on the next line. While numbers less than 10 are nearly always written out, for units of measurement, the numeral is used.

Numerals inserted – Lines 214 – 215

9. Line 223 (and elsewhere): Minor, but when I first read this, I thought that you meant number positive for larvae and then for pupae. However, on re-reading it, I realized that you meant all immature stage (i.e., both larvae and pupae) and then just for pupae. Would it help to change “immature stages” to “all immature stages” or possibly to change larval “immature stages” to “immature stages (i.e., both larvae and pupae)” the first time this is used? Note, I noticed that the definition of immature stages was given in Tables 3, 5, and 6.

“Immature stages (i.e. both larvae and pupae)” is inserted the first time it is used – Line 38

10. Lines 232-236: I am having a problem with these numbers. If 28.5% of all of the houses were positive for Ae. aegypti, how can the positivity rates be 60.4 and 39.6% for the rural and urban areas. I understand that 226 of the houses in the rural area were positive, but according to the previous sentence and the table, 683 houses were tested. Thus, shouldn’t the positivity rate for the rural area been 33.1%? The same comment applies to all of the other rates mentioned in this section.

These rates were calculated out of the total number of positive houses (i.e. 374). We have changed the denominator and recomputed the rates using the number of inspected houses in the setting and season - Lines 270 – 273.

11. Lines 247-248: Again, I am having a problem with these numbers. If 64.8% of the inspected water containers were positive, how could only 28.5% of the houses be positive? I am guessing that many of the houses actually had no water containers at all and that others had a lot of positive ones. You might want to clarify this.

Yes, we observed a number of positive houses with two or more positive water containers. A clarifying statement is added - Lines 286 - 288

12. Lines 249-250: Looking at Table 3, there appears to be 729 containers in the Rural area and 364 in the Urban area. Why were there 708 containers examined for all immature stages in both the Urban and Rural areas? Why is the denominator for pupae different than that for all immatures? To me, this sentence is saying that in rural areas, 471 of 708 containers were positive for immatures, yet according to Table 3, 471 of 729 containers were positive for immatures in the rural area. Please check all these numbers.

We have checked and changed the denominators from 708 and 318 to 729 and 364 for rural and urban settings, respectively. And the same denominators are used for immatures and pupae – Lines 289 - 293

13. Table 5: I know that the statistics are shown in Table 8, but it might be nice to indicate the statistically significant differences. For each of the parameters (volume, sun, vegetation, and organic), you could follow each of the percent positives with a letter. If they were followed by the same letter, then they would not be significantly different, but if followed by different letters, they would be significantly different. For example, for Organic matter, Yes, would be followed by an “a” and No would be followed by a “b.” For Sun exposure, both < half day and >half day would be followed by a “a,” and for Water volume, <5L would be followed by an “a,” 5L-20L followed by a “b,” and >20L followed by a “b.”

The statistically significant differences are indicated as suggested – Table 5 with a corresponding legend on lines 345 – 346
14. Line 321: Minor, but I don’t think that “Anopheles” has been established prior to this line.

Anopheles mosquito has been introduced in the introduction section - Lines 115 - 116

15. Line 336: In this section the authors present numerous statistics and confidence intervals for differences. However, numerous comparisons were made. Some of the CI barely don’t include 1 (e.g., line 345, for rural versus urban, the lower limit is 1.11). Is it possible that if these numbers were adjusted for multiple comparisons, that the CI would have included 1?

We repeated the analyses to account for effect of repeated observations on the outcome variable across settings overtime (random factor) based on reviewer #2’s comments and reproduced the results – Table 7 & 8 with the corresponding interpretation – Lines 384 – 403, and 438 - 444

16. Line 345 (and elsewhere): Yes, it may be 1.53 times higher odds, but is the hundredth place really meaningful? Consider rounding all of these to the nearest 10th. See also line 351. Is the thousands place at all meaningful here?

The ORs and IRRs and their corresponding CIs have been rounded as suggested - lines 384 – 401, 440 – 441 and Tables 7 & 8

17. Line 358-360 (and other places): The authors compared <5L and >20L multiple times, but never mentioned <5L vs 5-20L or 5-20L vs >20L. Based on Table 5, volumes of <5L should be significantly higher than both 5-20L and >20L, and 5-20L should not be significantly different than >20L. Is that correct?

In this study we organized and defined water volume levels as low (<5L), medium (5L – 20L), and high (>20L) (Table 1). In the statistical analyses the category >20L was set as a reference (statistical model requirement) where each of the other two categories was compared with it, i.e. <5L vs >20L, and 5L – 20L vs >20L.

To compare <5L vs 5L – 20L we applied Tukey’s HSD multiple comparisons test, the results of which are presented in the manuscript wherever water volume was significant – Lines 391 – 393 and 401 – 403.

18. Tables 8 and 9: The tables merely give the p-value for whether or not the two parameters are significantly different. Although it is explained in the text, the reader can’t tell from the tables which of the two is more likely to have immatures or pupae. Yes, it does appear that you are comparing the first listed against the second listed, but it never states that. Might it be worth a footnote to “Parameter” along the lines of “Likelihood that the first parameter produced more immatures or pupae than the second parameter.” Also, what about the comparison between <5L and 5-10L.

The footnote inserted on Table 7 and 8 as suggested – Lines 476 – 477 and 481-482.

The category 5-10Lwas not applied in this study. The included variables and their categories are described in Table 1.

19. Lines 416-417: Minor, but the number of immature Ae. aegypti stages if both larvae and pupae were present would be 2. The number of “immatures” is what you are examining. I must admit that this is a minor and the readers will understand, but technically, the answer is 0, 1, or 2 for the number of stages present.

We have omitted the confusing statement/legend on Table 8 and added clarifying texts on the column headings on table 7: presence of immature stages/pupae and on Table 8: abundance of immature stages/pupae

20. Line 422: Is it worth a short sentence here. We found HI and BI between 15 and 42 and between 17 and 106 for HI and BI, respectively, depending on season and location.

The sentence is inserted - Lines 489 - 490

21. Lines 452-455: Yes, I know that plastic containers contributed the most. However, was it because plastic containers were more attractive or a better habitat, or was it because there were so many more plastic containers. If you look at it slightly differently, percent of type of container that was positive for Ae. aegypti immatures, then the order is reversed, with plastic having only 65%, metal being 73%, and tires have a 82% positive rate for Ae. aegypti. This is almost a contradiction of lines 458-462.

We checked the numbers (percent of positive container by type) and deleted the contradictory statements – Lines 529 - 531

22. Line 464 (also 478 and 536): Again, mosquitoes do not “breed” in tires, their immatures develop there.

“Breeding sites/habitats” has been removed and replaced with the words “larval” or “immature” – Line 26 and throughout the manuscript

23. Line 478: Also, the trade in used tires is important as that is an excellent means of transporting Aedes eggs to a new location.

The suggested statement is added – Lines 549 - 551

24. Lines 498-499 and 550: why establish “ZAMEP” if you do not use it again. Either do not establish or use on line 55

“ZAMEP” is used on line 627

25. Line 552: Why establish “IDSR” if it is not used. Please check to see if ALL of the abbreviations established are actually used. If not, then please remove these unnecessary abbreviations.

“IDSR” deleted (Line 629) and all unused abbreviations removed

26. References: Please ensure that these are formatted according to PLoS NTD guidelines. Only the first word or proper nouns in a title should be capitalized. See reference 3 and others.

We have made corrections on references

**Reviewer # 1**

Recommendations:

1--Throughout, to be accurate, replace the term “breeding” habitat with larval habitat.

We have replaced breeding habitat with larval habitat throughout the manuscript

2--Line 380 – what type of vegetation in containers? Please indicate here, before you reveal the presence of algae in the Discussion.

Algae is added – line 387

3--Line 386 – be careful of saying borderline significant. It can be misleading. I prefer significant or not.

The statement is deleted, and results were rewritten - Lines 384 - 403 and 438 - 444

4--Line 498. Integrating Aedes control with malaria vector control is important. Also note this approach is consistent with IVM guidelines recommended by WHO.

We inserted “This approach is consistent with integrated vector management guidelines recommended by WHO” – Lines 574 -575

5--Line 518. Limitations. Also include as a limitation, the short duration of the study, thought it covered periods of the wet and dry season. Another limitation is rather short duration of in the field mosquito sampling during both wet and dry seasons.

The limitation is added – Lines 609 - 610

6--Aedes surveillance is important for any efforts in vector control. It will be very helpful if you give some guidance on how this can be done.

We have inserted a brief statement describing surveillance of *Aedes* mosquitoes – Lines 74 - 77

7--References 1, 10, 21 and more. Are there references available? Avoid url’s. The high percentage of url’s instead of references detracts from the quality of the manuscript.

Reference 1 is replaced with published journal article. We could not find other references for reference 10, 12, and 23. Nevertheless, these are the only remaining url citations in this article.

8--Figure 1, it is ok but why are the urban and rural sites so far disconnected? Normally, it is common to work in urban areas and select rural sites just outside the urban areas. Selection bias of sites is a major concern, not just in this study; entomologists typically choose sites where they expect to find the most mosquitoes. Was this the case here? More discussion is needed to provide a better rationale for your site selection.

The study sites were purposively selected as described in the methods section – Lines 178 – 179. This is acknowledged as a limitation and its rationale is explained on lines 596 – 600.

9- Mosquito identifications. It is ok to rear immatures and identify adults. Was this done even for first and second instars? Please clarify in the methods. It is oftentimes preferable to identify larvae, and let pupae emerge. This should be considered in the future.

Yes, all collected Aedes immatures including first and second instars were reared to adults and identified as clarified in the methods section – Line 225 - 227

In future studies, we will consider identification at larvae stage as advised.

10—Length of manuscript. The manuscript is long and should be shortened without loosing content. Suggest shortening 10 to 20%.

To reduce the word count, we have omitted lines 368 – 381 and submitted Table 7 as supporting information (S1 Table). In addition, since Tables 7&8 present complete sets of results, we have deleted the repetitious text in the results section = Lines 404 – 437, and 445 – 472.

**Reviewer #2:**

An interesting study assessing the traditional Stegomyia indices and typology of breeding sites in rural and urban areas in Zanzibar. Paper is well written but some clarification and additional analyses are needed before accepting for publication in PNTD.

Lines 42-43. How the productivity was assessed?

Productivity was assessed based on the abundance of *Ae. aegypti* pupae in a given water container as stipulated in the WHO operational guide for assessing the productivity of *Ae. aegypti* breeding sites (Reference No. 34).

We have added clarifying texts – lines 47 – 48 and 201 - 202

Line 66. Ae. Aegypti please correct it.

*Ae. aegypti* corrected – line 71

Material and Method
Which technic was used to select houses for inspection?

The inspected houses in each season were randomly selected using house IDs (using R software) from a sampling frame of all households in the respective *Shehia* provided by the local community leader (*Sheha*). This information is presented in the manuscript – lines 194 – 196.

Did the selected houses cover all the environments of the city?

Yes, except for the stone town area which was covered in our previous study (Reference No. 29). The urban study sites are located about 2.5 km from Zanzibar Stone Town at the outskirts of Urban district, representing the main urban environments of the Island.

It would be good to add a map indicating the houses selected. The same houses were prospected during the dry and raining season?

The houses were randomly selected from the full list of houses in each season. We acknowledge that a possibility of a house or container being sampled repeatedly in each setting was there. Therefore, we reanalyzed the data using Generalized Linear Mixed Methods (GLMMs) models to account for possible dependence between repeated measures across settings overtime. As such, to check whether adding random effect structure is justifiable, we fitted two candidate models, that is, a model only considering fixed effects and the other considering both fixed plus an observational level random effect, that is, a mixed model. The model with smallest Akaike Information Criterion (AIC) was considered best fit. This information is presented in the data analysis section – Lines 235 - 249

The number of pupae and larvae were counted?

Yes, in the insectary larvae were separated from pupae and counted as presented – lines 225 - 227

What was the rate of transformation of larvae into adults?

About 72% of the reared *Aedes* immatures emerged as adults. This statement is inserted – line 227 - 229

Why pupae based indexes were not calculated? It would have been good to count the number of people per house to assess the number of pupae per person.

We have calculated pupae per person index (PPI) - Lines 209 – 211, Table 2, Line 278, 298 – 299, line 301, and lines 490 – 491

Results
The difference between indices calculated (BI and HI) was significantly different according to the season or environment (urban vs rural)?

The traditional *Stegomyia* (HI, BI,) and pupae (PPI) indices were not statistically significantly different between seasons across settings. This information is added on lines 45 – 46, and 299 – 301.

What type of container was mainly found indoor?

Containers found indoor were mostly plastic and metal containers such as plastic tanks, steel/metal tanks, buckets, jerrycans, drums, basins/bowls, of different sizes with specific function or unused. Nonetheless, similar containers were also found outdoor. This information is added – lines 545 - 547

Table 6 Tanks instead of “tanks”.

Corrected

How authors did to differentiate the pupae of Ae. aegypti to others species?

We counted and reared all pupae and identified species after emergence as described in the materials and methods section – Lines 225 - 227.

Lines 427-428. It would be fairer to compare the mean number of immature stages per container or house rather than the overall abundance between rural and urban setting.

We agree to this comment, however, the manuscript is already too long such that the reviewer # 1 suggested a 10 - 20% reduction. Therefore, adding this information would have increased the text substantially.

Line 459. What could explain the propensity of Ae. aegypti to bred in plastic containers?
We speculate that this could be attributed to the fact that plastic containers were readily available in the environment mainly in the form of water storage containers and unmanaged waste. We recommend further studies to ascertain the influence (if any) of container material in attracting oviposition and development of immature stages. Lines – 526 - 533

Higher indices reported in this study are comparable with the case of other African cities?

There are studies in African region which reported similarly high Stegomyia indices. Statement and references are added – Lines 491 - 492

Have such high indices previously coincided with true outbreaks occurring?
To the best of our knowledge, this is the first study to document the *Stegomyia* indices in Zanzibar and currently no *Aedes* surveillance or control programs exist. Interestingly, no arboviral disease outbreak has been officially declared in the Island to date, despite studies that document evidence of previous or current infections as described in the introduction section– Lines 100 – 105
