# Supplementary material for: Epidemic risk of arboviral diseases: Determining the habitats, spatial-temporal distribution, and abundance of immature Aedes aegypti in the Urban and Rural areas of Zanzibar, Tanzania
Source: PLoS Negl Trop Dis. 2020 Dec 7;14(12):e0008949. doi: 10.1371/journal.pntd.0008949 (PMC7746278; doi:10.1371/journal.pntd.0008949)
Supplement: Attachment — Submitted filename: Response to Reviewers file1.docx [file pntd.0008949.s003.docx]

**Cover letter regarding revision of the manuscript entitled: “Epidemic risk of arboviral diseases: determining the habitats, spatial-temporal distribution and abundance of immature *Aedes aegypti* in the Urban and Rural areas of Zanzibar, Tanzania” submitted to PLoS NTD journal.**

We highly appreciate the additional comments, suggestions, and corrections made by the journal Editors, and would like to acknowledge their time and efforts spent on our manuscript. In this letter, we have point-by-point addressed the comments as received from the Editors. Our response is written in blue color font.

**Editor’s comments**

1. I had a hard time correlating the line number indicated in the response letter and the line number in the revised manuscript. For example, in the response letter it said, “The footnote inserted on Table 7 and 8 as suggested – Lines 476 – 477 and 481-482.” However, those footnotes were on lines 377-378 and 482-483. Similarly, in response to the comment about Dr. Mohammed’s work, the authors responded, “…which we refer in the revised manuscript (Ref 20), - Lines 102 - 103.” However, that was inserted on lines 99-100. Please check to ensure that the line numbers mentioned are accurate for the final, revised, clean copy.

We sincerely apologies for the line number miscorrelations between the previously submitted response letter and the revised manuscript. We assure you the line numbers indicated in this letter correlates with the revised manuscript, clean copy.

2.  Minor, but is it one space or two spaces between sentences? Either is fine but be consistent. I also noted some extra spaces between words. You might want to do a search for “space space” (obviously not the words) and remove the extra ones.

We have checked and removed extra spaces

3.  Line 43 and others: Again, as indicted earlier, the number of immature “stages” observed in a given sample is only 0, 1, or 2. You are not reporting the number of “stages,” but the number of immatures. I would remove “stages” throughout much of the manuscript. Note, “immature stages or pupae” is confusing. I believe that what you are looking at is all immatures (i.e., larvae and pupae) or pupae only. I would consider replacing the phrase, “immature stages or pupae” with “all immatures or pupae only” throughout the manuscript. Note that on line 228 you clearly indicated that “immature stages” indicates both larvae and pupae, but it still bothers me as you are not trying to measure the number of “stages,” but rather the number of “all immatures” or just the number of pupae. When you are just referring to immatures, i.e., line lines 37, 202, 214, 219, 228, 235, 273, etc. (i.e., where it does NOT say immature stage or pupae) then the use of “stages” is appropriate. However, the use of “stages” on lines 43, Table 3, 308, 317, Table 5, 336, Table 6, 398, 424,425, and 426 is not appropriate.

We have replaced immature stages with “all immatures” or “immatures” throughout the manuscript except on lines 210, 215, 231, 423 where it does not refer to immature stages and/or pupae

4.  Lines 255-257: Yes, the rate was much higher, but was it significantly different? Actually, it is at a p-value <0.0001, so you might want to add that the difference was highly significantly different. Same comment for wet versus dry.

We ran Chi-square tests and added the texts and statistics – lines 252 - 255

5.  Lines 301-306. Similar comment. Yes, more containers were identified indoors than outdoors, but was this difference significant?

A sentence is added lines – 299 - 300

6.  Line 321, Table 5: I just rechecked this table, and there are a number of problems.
a.  Did you really look for “immature stages and pupae?” Wouldn’t looking for immature stages include looking for pupae? I would change the title to either “…Ae. aegypti immatures in Zanzibar” or possibly to “…immature Ae. aegypti in Zanzibar.”

Table 5 title is corrected as suggested – line 320

b.  Unfortunately, the way the spacing is done makes it difficult to see which groups are being compared. For example, having “Organic Matter” cover two lines makes it look like Organic matter “Yes” is part of the Vegetation group. Similarly, by having Water volume taking up two lines, it makes Water volume “5L-20L” and “>20 L” look like they are being compared with Sun exposure “<½ day” rather than with Water volume “<5L.” Thus, because there are a lot of columns, it might be better to use a landscape (horizontal) layout for this table. You might also change “Exposed half a day or less” to “Exposed < half day” or even “Exposed <½ day” and “Exposed >½ day” to shorten that area.

We have changed the layout of the table to landscape and replaced “half a day” with ≤½ or >½ day as advised – Table 5

c.  The layout is also a little confusing. The first column of “Containers positive” is for all immature states, while the second one is for only those positive for pupae. But this is not clear. Could you make it “Containers positive for immatures” and “Containers positive for pupae?” Also, should it be “No. of immature stages (%2)” or “No. of immatures (%2)?” Remember, there are two stages, larvae and pupae, so the real number of stages is two, which is different than the number of immatures present. Delete “stages” and use “No. of immatures (%2).”

We have accordingly corrected the column titles and deleted “stages” – Table 5

d.  I just rechecked the statistics, and it appears that 156/433 (36%) is highly significantly different than 152/660 (23%) by a Fisher’s exact test (p < 0.0001) for organic matter. Similarly, <5L is NOT significantly different than >20L (Fisher’s exact test, p = 0.282). When I originally wrote this comment, I had not checked the statistics, so I was just illustrating how to write it. You need to go back through and actually check the statistics for each of these, and then in the footnotes indicated how you did it and what you mean by “statistically different.” For example, the footnote could be, “The categories followed by different letters (a, b) within a given parameter were significantly different (Fisher’s exact test, p < 0.05) and those followed by the same letter (a, a, or b, b) were not significantly different.” Remember, all data needs to be presented in the past tense.

We acknowledge the issue concerning correct presentation of the statistics in Table 5. Please note that following your previous comment regarding the inclusion of statistics, we decided to present the statistical results generated from GLLM for binary outcomes in Table 5 (also presented in Table 7). Our decision was based on the fact that the Chi-square/ Fisher’s exact test results did not align with those of the GLMM (as explained in our response to comment # 8 below). This is most notable, as you point out, in the case of water volume, where the difference between <5L and >20L was NOT significant using Chi-square/ Fisher’s exact test, but significant using GLMM.

We thought it might confuse some readers, if we presented divergent results for the same variables in separate tables, based on different statistical tests, hence decided to present the statistics of the GLLM, only.

With this in mind, we kindly ask for your advice on how best to approach this issue. Should we 1) show the Chi-square/Fisher’s exact statistics in Table 5, as you suggest in your most recent comment, 2) keep the currently shown GLLM statistics, or 3) omit statistics in table 5 altogether, keeping it strictly descriptive as in the original version, where statistical results were reserved for Table 7 and 8?

We very much appreciate your opinion and apologize for this lengthy response.

7.  Line 338, Table 6: To me, another important question would have been what percentage of the various types of containers were actually positive for immatures or pupae. If I calculated it properly, 65% (344/529) of the plastic containers were positive, 73% (172/235) of the metal containers were positive, 82% (134/164) of the tires were positive. It might be nice to add this, possibly in parentheses after the number positive, so for Plastic, under positive containers, it would be “344 (65%)” with a footnote on containers that said, “Number of containers positive (percent of that type of container positive for Ae. aegypti)

We have added percent of positive containers by type of containers within parentheses in Table 6 as suggested and a corresponding footnote on line 338

8.  Lines 357-360: I ran both a Chi-square and a Fisher’s exact test on the <5L vs >20 liter, and the p-values were >0.28 as indicated above. Please check your statistics.

We agree with your observation. In this section (lines 346 – 368) the reported statistics were produced by GLMMs and not Chi-square/Fisher’s exact test because our aim was to determine predictors of presence and abundance of Aedes aegypti immatures for which the GLMMs were considered most appropriate. Further to our response on comment # 6d above, we strongly believe that the statistics obtained from Chi-square/Fisher’s exact (or any univariate tests) are not comparable to those produced by multilevel (or mixed) models. For instance, GLMMs determine the extent to which the covariates can predict the likelihood of an outcome, while accounting for possible dependence between repeated measures commonly observed in cross-sectional or longitudinal surveys.

Please note that we did not carry out any initial univariate analysis to select significant predictors, as we believe that applying univariate tests to guide selection of significant variables for subsequent analysis in multiple regression or mixed models can be misleading. Notably, a variable may show significant effect in a univariate regression test (or Chi-square/Fisher’s exact test), but no significant effect on the same outcome when analyzed in a multiple regression model and vice versa. This regression analysis “paradox” is clearly explained by Feng et al. 2016 [1].

In our case, neither exploratory Chi-square nor univariate regression tests showed significant effect of water volume <5L vs >20 on presence of immature Aedes aegypti. However, water volume was significant when entered into the mixed model (Table 7).

9.  Line 447: Minor, but “as it present an…” should be “as it presents an..”

Corrected Line - 440

10.  Line 465. Yes, Anopheles was established in the introduction, but that does not establish “An.” The first time you mention an Anopheles species, you need to write out Anopheles, or in this case, “Anopheles gambiae.”

Anopheles gambiae is written in full – line 458

11.  Line 563, Ref 11: Dengue Virus Type 1 should be “Dengue virus type 1…” as only the first word and proper nouns should be capitalized.

Corrected – Ref 11

12.  Supplemental table: I just wanted to confirm that out of the 13,677 Aedes larvae captured and identified, 13,671 were Ae. aegypti. That is very impressive

Yes, the numbers are correct. We were amazed ourselves when we found such high numbers of Aedes with predominantly aegypti sp. This might be attributed to the fact that neither Aedes surveillance nor control program has ever been implemented in the Island to date.

Reference cited

[1]. Feng G, Peng J, Tu D, Zheng JZ, Feng C. Two paradoxes in linear regression analysis. Shanghai Arch Psychiatry. 2016;28: 355–360. doi:10.11919/j.issn.1002-0829.216084
