# Supplementary material for: Epidemic risk of arboviral diseases: Determining the habitats, spatial-temporal distribution, and abundance of immature Aedes aegypti in the Urban and Rural areas of Zanzibar, Tanzania
Source: PLoS Negl Trop Dis. 2020 Dec 7;14(12):e0008949. doi: 10.1371/journal.pntd.0008949 (PMC7746278; doi:10.1371/journal.pntd.0008949)
Supplement: Attachment — Submitted filename: Response to Reviewers file2.docx [file pntd.0008949.s004.docx]

**Cover letter regarding revision of the manuscript entitled: “Epidemic risk of arboviral diseases: determining the habitats, spatial-temporal distribution and abundance of immature *Aedes aegypti* in the Urban and Rural areas of Zanzibar, Tanzania” submitted to PLoS NTD journal.**

We highly appreciate the additional comments, suggestions, and corrections made by the journal Editors, and would like to acknowledge their time and efforts spent on our manuscript. In this letter, we have point-by-point addressed the comments as received from the Editors. Our response is written in blue color font.

**Editor’s comments**

1. Lines 272-277: You wrote that the positivity rate for containers for immatures was significantly higher in the wet as compared to the dry season, but never mentioned that the rate was or was not significantly different for pupae (it is obviously not significant, but you never said so. Note, don’t merely write “These rates were not significantly different p > 0.05.” Calculate and indicate the actual p-value. Note, if the value was 0.06, it would not be significant, but given that the immatures were significant, this would indicate that the difference in pupae was likely to be real as 0.05 is an arbitrary cut off value. However, in the current case, I believe that the p-value is 0.94, by a Fisher’s exact test and clearly not significant. Also, it is interesting to note that the number positive for pupae in the wet season, 236, is again nearly three times greater than the number positive for pupae in the dry season, 82. This difference in number positive is not due to there being a higher positivity rate in the wet season, but due to there being nearly three times as many containers examined in the wet season. Thus, the initial sentence, “More than three times greater numbers of containers were positive for immature Ae. aegypti in the wet (N = 543/814, 66.7%) compared with dry (N = 165/279, 59.1%) seasons…” is a little misleading. The huge difference in numbers positive is mostly due to a much larger sample size in the wet season.

- We added a statement “However, the difference was not statistically significant (p=0.90)” – Line 275
- We deleted the words “More than three times greater” and rephrased the sentence as “Higher numbers of containers were positive for immature *Ae.aegypti*…..”……… Line 271

2.  Line 300: As mentioned above, rather than merely write “(p > 0.05),” please put the actual p-value here any anywhere else only “> 0.05” is used.

The actual p-value (p = 0.10) is added – line 300

3.  Lines 300-302: Were these differences in container types significant?

We added a statement “Nevertheless, no significant difference was found in the rates of immatures positive containers between discarded/other water receptacles (N = 257/416, 61.8%) and water storage (N = 214/313, 68.4%) (p = 0.07) in the rural setting. Likewise,……” - Lines 303 – 307 and Lines 318 - 322

Please note that, in the statistical analyses, due to smaller number of containers, the category other water receptacles was merged with discarded/unused containers.

4.  Table 5:
a.  Yes, I understand the difference and advantages of both GLMM and either a Fisher’s exact test or a Chi-square test, but I am still bothered by the apparent significant difference in Table 5 of “<5L” and both “5L-20L” and “>20L.” As we both noticed, these are not even close to significant by a univariate test. Are containers <5L really significantly more likely to contain immatures than larger containers or not? Looking at Table 5, this difference is “significant” only for the all immatures, but even close to significant for the pupae, yet for the <5L containers, the percent of the immatures positive (59%) was identical to the percent of the of the pupae positive (59%). I think that the “significant” difference by GLMM was due to chance. However, I will leave how to present the statistics up to you.
b.  Minor, but in footnotes 2 and 3 for Table 5, it says “immatures or pupae.” Because pupae are immatures, this is not correct. How about, “…immatures for each category…” and “…immatures within each category?” Note, in footnote 1 you wrote that “immatures” means larvae plus pupae.

We acknowledge your concern regarding the statistical results of water volume levels. In order to avoid confusion, we have decided to remove the variable water volume from our GLMM models and instead report water volume in Table 5 only while indicating Chi-square statistics in Table 5 as per your previous comment

Please note that, we have fitted new models omitting water volume as predictor and we accordingly revised the results on Table 7 & 8 and lines - 357 – 370 and made relevant changes in the abstract lines 43 – 44, and throughout the manuscript.
